# Excessive Lateral Trunk Lean in Patients With Cerebral Palsy: Is It Based on a Kinematic Compensatory Mechanism?

**DOI:** 10.3389/fbioe.2019.00345

**Published:** 2019-11-19

**Authors:** Roman Rethwilm, Harald Böhm, Chakravarthy U. Dussa, Peter Federolf

**Affiliations:** ^1^Orthopedic Children's Hospital Aschau, Aschau im Chiemgau, Germany; ^2^Department of Sport Science, University of Innsbruck, Innsbruck, Austria

**Keywords:** 3D gait analysis, gait pattern, principal component analysis, trunk control, Duchenne gait, motor function, compensatory strategy, cerebral palsy

## Abstract

**Introduction:** Excessive lateral trunk lean is a commonly observed gait deviation in children with cerebral palsy (CP), with implications for energy expenditure and the development of back pain. While the trunk lean toward the stance leg is widely interpreted as a compensatory strategy to unload the hip, in CP the relation to hip abductor muscle strength is only weak. Therefore, other mechanisms may play a role in the prevalence of excessive trunk lean in CP, or it could be a primary motor function deficit.

**Research Question:** Is the excessive lateral trunk lean in patients with CP part of an underlying biomechanical mechanism?

**Materials and Methods:** Patients with bilateral CP (*N* = 255; age 13.6 ± 6.6 years) were retrospectively included and divided into a group with (*n* = 174) and without (*n* = 81) excessive lateral trunk lean. Ten lower-extremity joint angle waveforms were analyzed using a principal component analysis (PCA) to identify patterns of correlated deviations from average angle waveforms. Binary logistic regressions were performed to determine the discriminative capacity of the identified patterns.

**Results:** The PCA identified correlated kinematic patterns, with lower-order patterns showing more common gait pathologies, such as torsional malalignments and crouch gait pattern. Within five patterns, significant (*p* < 0.0025) group differences were identified. Interestingly, the trunk lean was not always distinctive in these patterns and despite the significant differences their effect sizes were small. The logistic regression was unable to reliably classify patients based on their trunk lean patterns.

**Discussion:** The current study identified multiple trunk lean-related patterns, however, excessive trunk lean was not attributable to a distinctive CP related gait pathology or to a specific compensatory strategy. Overall, the results do not support the hypothesis that excessive trunk lean is part of a biomechanical mechanism. Therefore, it seems more likely that excessive lateral trunk lean is based on other disease specific dysfunctions, influenced by the severity of the disease.

## Introduction

An excessive lateral trunk lean is a commonly observed gait deviation in patients with cerebral palsy (CP) (Attias et al., 2015), with a prevalence as high as 72% in children with CP (Klum et al., 2015). This excessive trunk lean has been shown to result in an increased cost of locomotion (Salami et al., 2017) and may also contribute to the development of back pain, which is one of the most common pain sites in adults with CP (Opheim et al., 2009).

From a clinical perspective, an increased lateral trunk lean is widely viewed as a compensatory mechanism for hip abductor muscle weakness (Schmid et al., 2013), also known as Duchenne gait. This mechanism describes the inclination of the trunk toward the ipsilateral stance leg, which shifts the center of mass (COM) laterally and reduces the hip abduction moments significantly (Salami et al., 2017).

It is often challenging to identify the cause of an observed gait deviation and to determine whether the nature of a specific abnormal pattern is a primary feature of the disease or rather a compensatory strategy adopted to cope with an underlying gait problem (Schmid et al., 2013). Identifying a mechanism as primary or compensatory has far-reaching implications. Not knowing the underlying source of an abnormal movement pattern could result in untreated primary pathology or, even worse, in unnecessary treatment of a compensatory mechanism that would resolve when the primary pathology is addressed directly.

The lateral trunk lean in CP gait is a case where the source is not as clear and apparent as in some other gait phenomena. For example, compared to the name-giving Duchenne muscle dystrophy, studies in CP found only weak—yet significant—correlations with hip abductor muscle strength (Krautwurst et al., 2013; Klum et al., 2015). This indicates that muscle weakness contributes to the occurrence of an excessive lateral trunk lean, but also suggests that other factors may play a role or could possibly be more prevalent. Accordingly, some researchers suspected further contributing causes, such as bony deformities (Salami et al., 2017) or an underlying primary motor control deficit (Heyrman et al., 2014).

To gain further insights into potential underlying mechanisms for an excessive trunk lean, the current study explored if excessive trunk lean is part of a kinematic movement pattern that can be frequently observed in the gait of CP patients. To determine kinematic movement patterns, we applied a principal component analysis (PCA), a statistical method that identifies correlated patterns (PC-eigenvectors) in multi-dimensional data (Daffertshofer et al., 2004; Eskofier et al., 2013; Robertson et al., 2014). Furthermore, a score for each pattern and patient is computed, indicating the extent to which each individual patient exhibits a particular pattern. For such a pattern to represent a functional mechanism, we postulated three criteria: (1) excessive trunk lean needs to be a part of the pattern. (2) If CP patients are classified into a group showing excessive trunk lean (eTL) and a group non-excessive trunk lean (nTL), then we expected to find significant differences and medium or high effect sizes for the scores that patients in these groups receive. (3) We considered that there could be more than one mechanism that produces excessive trunk lean as part of its kinematic pattern, however, we expected that based on the scores subjects receive for these patterns, it should be possible to successfully classify the subjects into the eTL and nTL groups.

We postulated that if gait patterns can be found that satisfy these three criteria, then causative biomechanical relations can be established, that are indicative for the underlying origins of an excessive trunk lean. The absence of such patterns, in turn, would suggest other disease related deficits of the trunk control that are not correlated with other deviations in the kinematic movement pattern of CP gait.

In summary, the aim of the current study was to explore correlated patterns in the kinematic variables that characterize CP gait. We hypothesized that patterns can be found that satisfy the three postulated criteria, which would suggest that excessive trunk lean is part of a kinematic pattern caused by an underlying biomechanical mechanism. Understanding the mechanisms related to an excessive trunk lean and whether these are primary or compensatory could aid in the clinical decision-making and improve the management of CP.

## Materials and Methods

Patients with bilateral CP who were at least 5 years of age were retrospectively included from the database of the gait laboratory, which were measured between 2009 and 2018. Written consent was provided for research purposes by the patients and the study was approved by the ethics commission of the local medical association. Inclusion criteria was a Gross Motor Function Classification System (GMFCS) level I or II; accordingly, patients were able to walk freely without assistive devices or help. Excluded were obese patients according to the age-dependent body mass index thresholds suggested by the WHO (de Onis and Lobstein, 2010) and patients with documented spine deformities.

Patient data included instrumented 3D gait analysis data, where the kinematics had been captured with an 8-camera Vicon MX system at a sampling rate of 200 Hz. Joint angles were computed based on the Vicon Plug-In gait model including the trunk. All patients walked barefoot at self-selected speed along a 13 m walkway and at least 3 consistent step cycles needed to be present.

**Figure 1:**
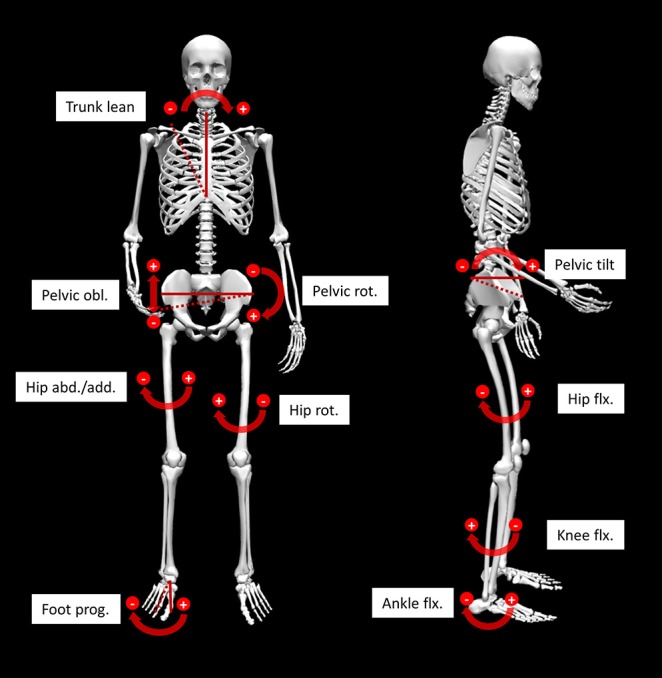
Illustration of the 10 analyzed angles. Angles referring to the global reference frame are indicated by solid and dashed lines. Other angles are referenced to the more proximal segment. obl, obliquity; rot, rotation; abd/add, abduction/adduction; flx, flexion; prog, progression.

**Figure 2:**
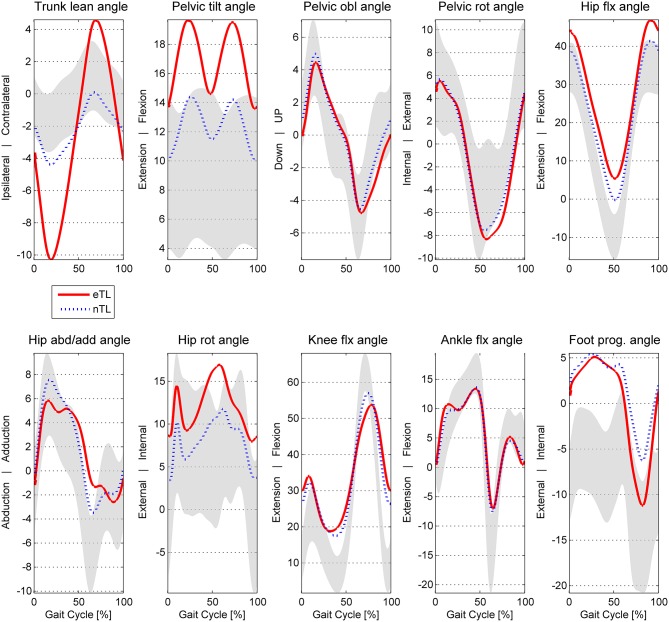
Angle waveforms averaged for the excessive eTL (excessive trunk lean) and nTL (trunk lean <3SD of norm) groups of CP patients. In gray: typical developed ± 1SD. obl, obliquity; rot, rotation; abd/add, abduction/adduction; flx, flexion; prog, progression.

For the analysis mean angle waveforms of the upper body and of the lower limb of the more pronounced side (greater lateral trunk lean) were processed. In detail, 10 angles were included: trunk lean and the 9 angles of the Gait Profile Score (GPS) of the lower extremity (Baker et al., 2009) (seen in Figures 1, 2), that represent clinically relevant joint angles in patients with CP. Furthermore, the GPS was facilitated as a descriptive measure to assess severity differences.

For further analysis the patients were divided into patients with and without an excessive lateral trunk lean (eTL and nTL, respectively), where “excessive” was defined as the trunk lean range of motion (ROM) angle exceeding 3 SD from a typically developed norm collective (TD; *n* = 24). The ROM was chosen over the maximum lateral trunk lean to account for spine deformities resulting in a constant lateral side bending. The 3SD cutoff was chosen based on the small TD trunk lean standard deviation and a lower cutoff would be hardly visible. The resulting threshold to classify for the eTL group was a lateral trunk lean ROM exceeding 8.4° (TD ROM + 3SD).

To identify patterns of correlated deviations from average kinematic angle waveforms, the principal component analysis (PCA) was facilitated. The PCA has already been successfully used in different contexts in CP, for example, to identify CP-related gait pathologies (Carriero et al., 2009a,b), to study multi segmental gait deviations (Zago et al., 2017) or to evaluate post-operative changes after multilevel surgery (Steppacher et al., 2018).

The input data for the PCA were the time normalized angle waveforms, each angle consisting of 101 discrete points, concatenated to a vector of 1,010 columns. Each gait cycle of every patient contributed a new row for the PCA input matrix. This approach has two advantages: firstly, it allows the systematic identification of kinematic gait patterns [principal components (PCs)] within and between the 10 angle waveforms, and secondly, a score is generated for each patient, expressing the coincidence of the patients movement pattern with each PC (Daffertshofer et al., 2004; Federolf et al., 2013). The individual PC-scores can then be used to further investigate the group differences between patients exhibiting an excessive trunk lean and patients who are not.

As part of the further investigation of the PC-scores, *t*-tests were used to identify group differences within the kinematic gait patterns and the standardized mean differences effect size Hedges's g (d) with 95% confidence interval (CI) were calculated. Due to multiple testing, the alpha error was conservatively adapted with the Bonferroni correction (alpha level *p* < 0.0025). Further, the PC-scores with significant group differences were facilitated to investigate classification rates using binary logistic regressions. This last step was used to verify that the identified kinematic patterns are distinctive for a lateral trunk lean mechanism since this would result in high classification rates. We considered the third criterion of the compensatory hypothesis for excessive trunk lean to be satisfied, when the classification rates exceeded the proportional chance criterion (PCC) for logistic regressions. For the analysis MATLAB (MathWorks Inc., Natick, USA) was used for the PCA and group comparisons and SPSS Statistics (IBM Corp., USA) for the binary logistic regression. For the skeletal joint angle visualization (Figure 1) OpenSim was facilitated (Delp et al., 2007; Seth et al., 2018).

## Results

For the current study *n* = 255 patients met the inclusion criteria (mean age 13.6 ± 6.6 years, 155 males 100 females). Of these patients *n* = 174 (68%) exhibited an excessive lateral trunk lean within their gait pattern while *n* = 81 (32%) stayed below the cutoff of 8.4° trunk lean ROM (TD ROM + 3SD). In terms of anthropometrics the two groups were very similar (Table 1). As for the functional status, the excessive trunk lean group (eTL) had a larger proportion of patients rated as GMFCS II (82.2%), compared to 64.2% in the normal trunk lean group (nTL). This was also reflected by the GPS, where the eTL showed higher deviations from the norm. The differences in GPS were highly significant (*p* < 0.001) when comparing the eTL and nTL groups.

**Table 1:** Anthropometrics and severity.

**Group characteristics**	**eTL (*n* = 174)**	**nTL (*n* = 81)**	***p*-value**
Age (years)	13.6 (6.7)	13.6 (6.4)	0.80
Height (cm)	148 (19)	148 (16)	0.62
BMI (kg/m^2^)	18.4 (3.3)	18.5 (3.4)	0.99
GPS (°)	9.4 (2.1)	7.9 (2.8)	** <0.001**
GMFCS I (%)	17.8	35.8	<0.001^*^
GMFCS II (%)	82.2	64.2	

* *χ^2^-test*.

*eTL, excessive trunk lean; nTL, non-excessive trunk lean*.

*Significant group differences in bold*.

Joint angle waveforms averaged over the two groups are displayed in Figure 2. The mean maximal trunk lean ROM was 16.68 ± 6.4 in the eTL, nTL 6.63 ± 2.8 and 4.18 ± 1.4 in TD.

**Figure 3:**
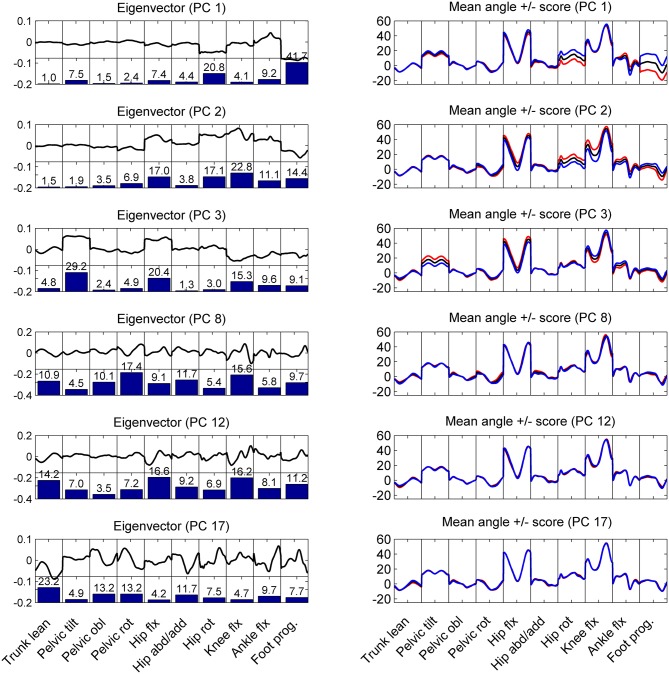
Left column: the graphs show the shape of the eigenvectors (thick black line) and how much in % each body angle contributed to the eigenvector (bar plots) for the first and significant PCs. Right column: mean angle waveforms (black line) and how these waveforms change for positive (blue) or negative (red) scores in the corresponding PC component. These graphs show that PC 1 and PC2 represent patterns of correlated variations in the kinematics of the patients that do not affect the trunk lean (first panel in each graph). However, PC 3, 8, 12, 17 are examples of variations in the kinematic patterns that influence (correlate with) the lateral trunk lean: the higher a patient scored on PC3, the more trunk lean did the patient show.

Within the between-patient variations in the kinematic variables the PCA identified correlated and mutually orthogonal patterns (Figure 3). The lower-order patterns, specifically PC-vectors 1 and 2 represented CP-gait deviations that did not change over the gait cycle, such as internal rotation malalignment of the lower extremity (PC1) and crouch gait (PC2). Higher-order PCs increasingly represented phase-dependent systematic gait deviations, including the trunk lean (PC8, PC12, PC17). More specifically, PC1, accounting for 31% of variability, showed a kinematic pattern dominated by the rotational malalignments of the lower extremity, expressed particularly as correlated deviations in hip rotation and foot progression. The contribution of these angle waveforms is visualized by the bar plots in Figure 3, showing that 63% in contributed by hip rotation and foot progression. The positive correlation between hip rotation and foot progression is visualized by the Eigenvector graph being negative in both cases. This means that the foot progressing increases with increased hip rotation or vice versa. Whether the correlation is positive or negative can also be seen in the right column mean angle visualization indicated by the colored lines. Is the colored line in both cases above or below the mean angle, the correlation is positive. Is the colored line opposite sides, the correlation is negative. PC2 depicted a combination of hip and knee flexion, coupled with hip rotation and foot progression pronounced during swing phase. PC3 expressed between-patient variations in pelvic tilt coupled with variations in hip and knee flexion.

**Figure 4:**
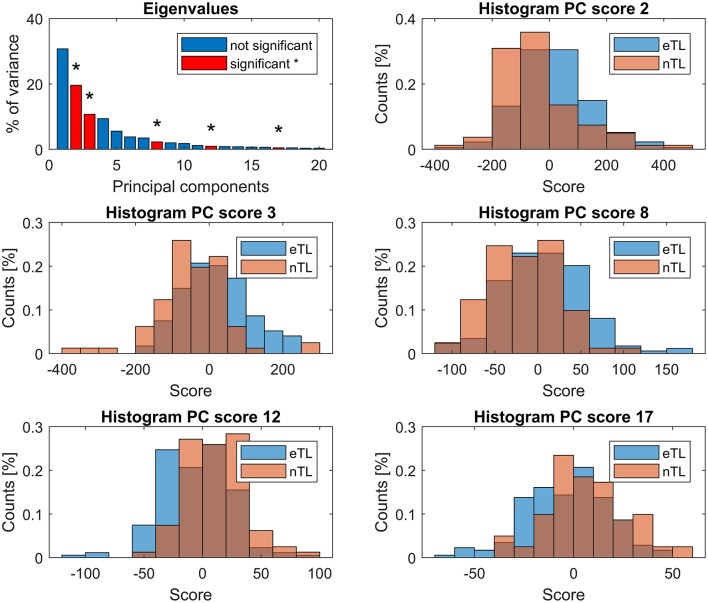
First panel: eigenvalues, showing the explained variance by each PC. PCs whose scores differed significantly (*p* < 0.0025) between the eTL and nTL groups are displayed in red and marked with *. Remaining panels: histograms showing the group score distribution and their overlap.

Within the first 20 PCs, 5 kinematic patterns with significant differences (*p* < 0.0025) between the scores of the eTL and nTL groups were identified (Figure 4). The trunk lean was distinctive in 4 of these kinematic patterns (PC: 3, 8, 12, 17), whereas PC2, while being significantly different between the eTL and nTL groups, did not entail the trunk lean within its kinematic pattern (Figure 3). Overall, the first 20 PCs explained 96% of the variability between patients. The PC components that differed between groups together explained 34% of the variability. The 4 kinematic patterns that contained trunk lean as part of their pattern, as visible in PC 3, 8, 12, 17 in Figure 3, accounted for 14% of the between-patient variability. However, although highly significant group differences were found within the scores of the different kinematic patterns (PCs) a substantial overlap of the score distributions of the eTL and nTL groups were observed (Figure 4), corresponding to small effect sizes for the group differences (Table 2).

**Table 2:** Overview of the patterns (PCs) with significant score group differences.

**PCs**	***P*-value**	**Effect size (ES)** **(d)**	**ES 95% CI**	
			**Lower**	**Upper**
2	<0.001	0.20	0.08	0.33
3	<0.001	0.29	0.18	0.40
8	<0.001	0.24	0.13	0.35
12	<0.001	0.28	0.17	0.38
17	<0.001	0.22	0.10	0.33

Individually, none of the PCs had sufficient predictive power in a logistic regression model (Table 3)—particularly nTL patients were falsely classified at a very high rate. The binary logistic regression model combining PC 2, 3, 8, 12, and 17 revealed a significant association between the lateral trunk lean and the 5 significant PC scores [*X*^2^_(5)_ = 96.196, *p* < 0.001] and explained 44% of variance (Nagelkerke *R*^2^). The overall predictability of the model was 81.2% (Table 3), however, whereas eTL patients were correctly predicted by the logistic regression at a rate of 89.7%, nTL patients were predicted correctly at rates of 63.0%. For the given group sizes—n(eTL) = 174; n(nTL) = 81—the proportional chance criterion (PCC), necessitates a nTL classification rate of at least a rate of 72% for an acceptable accuracy of an 25% improvement over a by-chance classification.

**Table 3:** Logistic regression models for the individual significant scores and for the combined model, with odds ratios (OR) and classification results for the excessive (eTL) and non-excessive (nTL) trunk lean groups.

**PCs**	***P*-value**	**OR** **(95% CI)**		**Classification rate (%)**	
				**eTL**	**nTL**
2	0.002	0.995 (0.993–0.998)		97.7	3.7
3	<0.001	1.010 (1.006–1.014)		93.7	14.8
8	<0.001	0.982 (0.973–0.990)		96.0	14.8
12	<0.001	1.032 (1.018–1.046)		95.4	12.3
17	0.001	0.968 (0.953–0.984)		96.6	11.1
Combined model 2 + 3 + 8 + 12 + 17		Overall		89.7	63.0
		Combined		81.2	

## Discussion

The current study analyzed the between-patient variability in CP gait patterns with a focus on lateral trunk lean. We hypothesized that excessive trunk lean might be part of a functional kinematic mechanism, and postulated that then we should find patterns in which trunk lean is correlated with other deviations in kinematic variables (criterion 1), in which patient groups with excessive (eTL) or normal trunk lean (nTL) would score significantly and substantially (effect size) different (criterion 2), and which would together allow to reliably classify individual patients as belonging to the eTL or nTL group (criterion 3). The PCA analysis conducted in the current study successfully identified several patterns (PC 3, 8, 12, 17) in which trunk lean was correlated to changes in other kinematic variables (criterion 1), and we found that subject groups scored significantly different on these four PCs (criterion 2a). However, we found that the score distributions of the two patient groups on all four PCs largely overlapped, corresponding with small effect sizes for the group differences (criterion 2b not satisfied). Furthermore, we found that the logistic regression model—despite yielding a significant association between the PC scores and excessive trunk lean—was not reliably able to classify patients into the correct group. Particularly patients with a normal trunk lean were to a high percentage falsely classified as eTL patients (criterion 3 not satisfied). Overall, these findings suggest that an excessive lateral trunk lean in CP is not based on a kinematic compensatory mechanism but, in a large fraction of the patients, more likely the result of other motor functional deficits (Panibatla et al., 2017).

Interestingly, no significant differences between eTL and nTL were found within the first kinematic pattern (PC1) entailing rotational malalignments of the lower extremity. Therefore, it can be concluded that lateral trunk lean mechanics are unaffected by these rotational malalignments. This finding stands in contrast with the assumption that hip internal rotation is a compensatory mechanism for hip muscle lever arm dysfunctions of the often anteverted hip in CP (Arnold et al., 1997). Since hip muscle weakness accounts, at least to some degree, for the prevalence of a trunk lean pattern (Krautwurst et al., 2013), a connection between hip internal rotation and trunk lean would have been plausible but could not be established in the current study. Another study found a significant negative correlation between hip rotation and foot rotation (Gaston et al., 2011) and propose the internal hip rotation as result of a distal foot external rotation. While the PC1 pattern contrarily shows a positive correlation of hip rotation and foot progression, the PC2 pattern shows the, by Gaston et al. proposed, functional relation of internal hip rotation and foot external rotation, combined with crouch gait characteristics, due to the lever arm dysfunction of the plantar-flexion knee-extension couple (Sangeux et al., 2015). The fact that the PCA revealed two different rotational patterns, the presence of two individual mechanisms is likely.

In the 2nd pattern (PC2) changes in trunk lean angle was not part of the PC-vector, however, the eTL and nTL groups scored significantly different when projected onto this pattern. The observation that excessive trunk lean was not part of this pattern, implies that more severely affected patients were more likely to also show excessive trunk lean (Attias et al., 2015; Swinnen et al., 2016), without excessive trunk lean itself being correlated to the PC2 gait variables. Other studies also found an increased trunk lean with increasing impairment (Attias et al., 2015), which is in line with our findings of more severe gait deviations within the eTL group expressed by the highly significant GPS differences. Additionally, the proportion of patients rated GMFCS II was about 20% higher in the trunk lean group, which further corroborates the severity explanation and was also observed in other studies (Swinnen et al., 2016).

The other 4 kinematic patterns (PC 3, 8, 12, 17) that differed significantly between eTL and nTL groups did not appear to show clear functional mechanisms. This could be partly due to the fact that the trunk lean strategy appears to be present in a variety of gait pathologies. The heterogeneous patient groups with diverse combinations of different gait pathologies is likely to result in multiple patterns, of which some will also contain trunk lean as part of their pattern. However, this does not imply a causative nature of the trunk lean, describing functional patterns. What can be said is that patients showing a particular combination of angle deviations (specific for the pattern), usually also exhibited an excessive lateral trunk lean. Since the identified patterns do not entail clear functional mechanisms, such as relations between frontal plane trunk, pelvis and hip kinematics, it seems plausible to conclude that neither a universal trunk lean mechanism exists, nor that a specific trunk lean strategy exists that is attributable to certain CP related gait pathologies. In a clinical sense, these findings support a multifactorial cause of a lateral trunk lean, implying that there is no single solution for addressing or correcting excessive trunk lean.

### Limitations

To provide further insight into how the results of the current study can be interpreted, some reservations should be mentioned and kept in mind.

One of these limitations is the circumstance that the gait speed was self-selected and different velocities result in altered angle patterns (Schwartz et al., 2008), including altered trunk kinematics (Thummerer et al., 2012). Despite the gait speed being not significantly different between the groups, it may still have some effect on the PCA results. PCA has been shown to be able to detect running speed differences (Maurer et al., 2012).

Arm movement was not measured. Arm movements in CP, however, may influence the trunk kinematics and might entail further information for the prevalence of an excessive trunk. Children with CP often show flexed elbow positions and increased shoulder abduction, which might be caused by spasticity but is also thought as compensatory strategy for balance and guarding purposes (Galli et al., 2014). Despite these general influences of arm movements on posture, the measured trunk lean should be largely unaffected, since the Plug-In gait model uses only the thorax markers, without the shoulder markers to calculate the trunk lean.

Furthermore, excessive trunk lean was defined as exceeding 3SD from norm. This definition is not based on a clinical classification of a certain degree of trunk lean being pathological. Hence, defining a meaningful cutoff will require further research and insight into the underlying mechanisms of excessive lateral trunk lean in CP.

## Conclusion

The PCA was able to identify kinematic patterns that were significantly related to the lateral trunk lean based on the group differences. However, despite these findings, a clear kinematic mechanism leading to excessive trunk lean was not found. The current study does not provide conclusive evidence against a kinematic compensatory mechanism. However, the absence of such patterns makes it more likely that excessive lateral trunk lean in CP could be the result of disease related motor functional deficits. More research is necessary to clarify this issue. Our study does provide evidence that rotational malalignments present independently of the trunk lean and that the prevalence of an excessive lateral trunk lean in CP depends on the severity of the disease.

## Data Availability Statement

The datasets generated for this study will not be made publicly available. Written consent included only the instituional use of the data.

## Ethics Statement

Ethical review and approval was not required for the study on human participants in accordance with the local legislation and institutional requirements. Written informed consent to participate in this study was provided by the participants' legal guardian/next of kin.

## Author Contributions

All authors listed have made a substantial, direct and intellectual contribution to the work, and approved it for publication.

### Conflict of Interest

The authors declare that the research was conducted in the absence of any commercial or financial relationships that could be construed as a potential conflict of interest.
